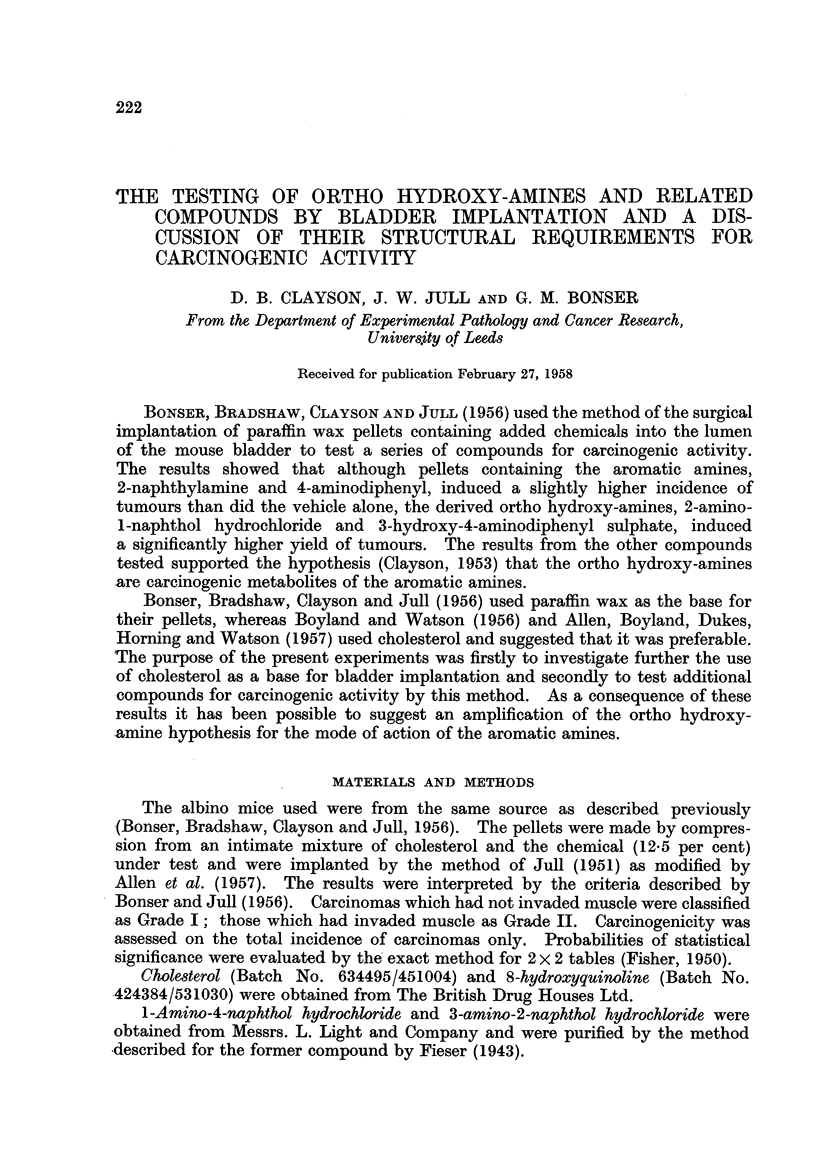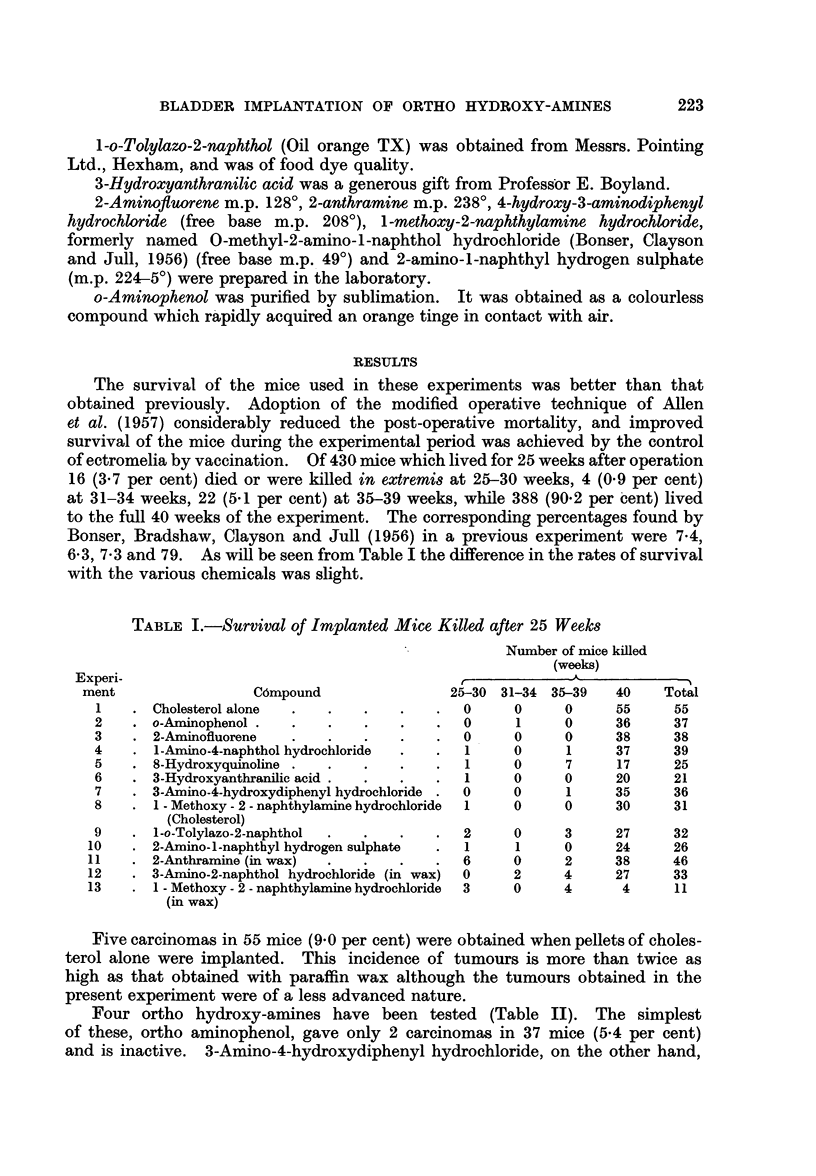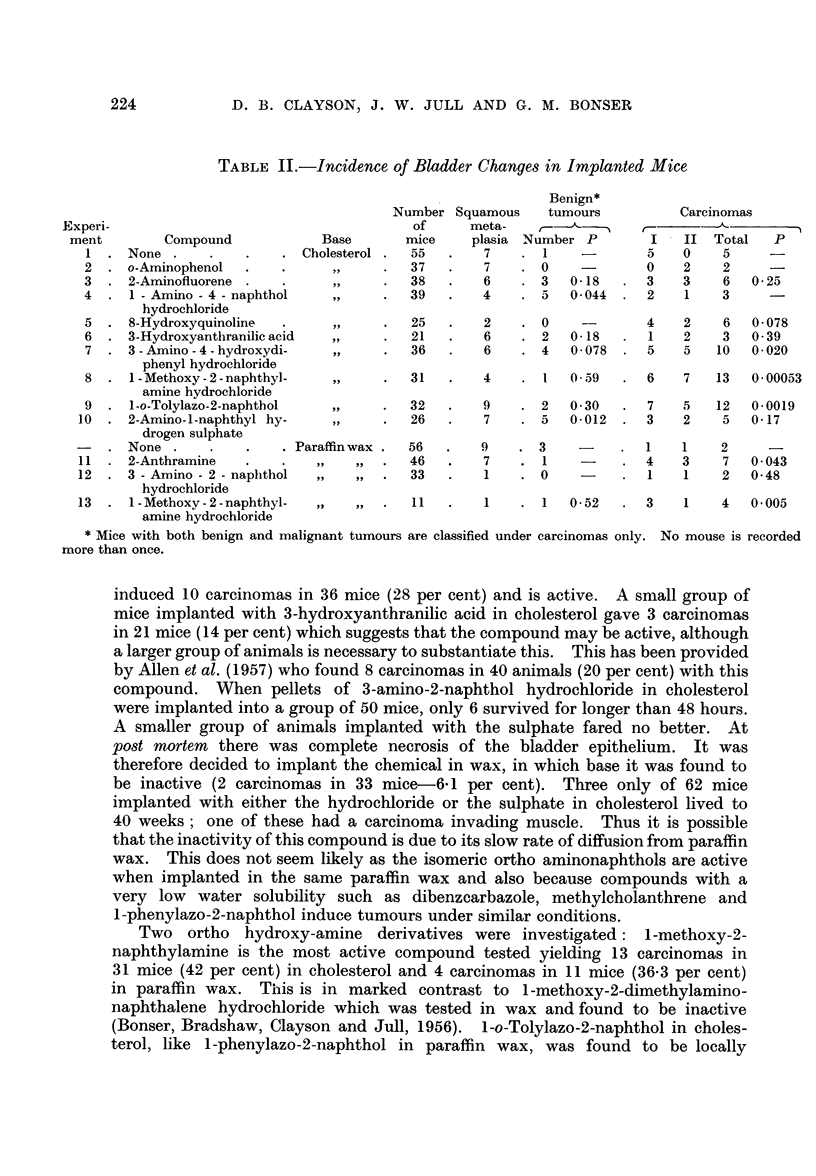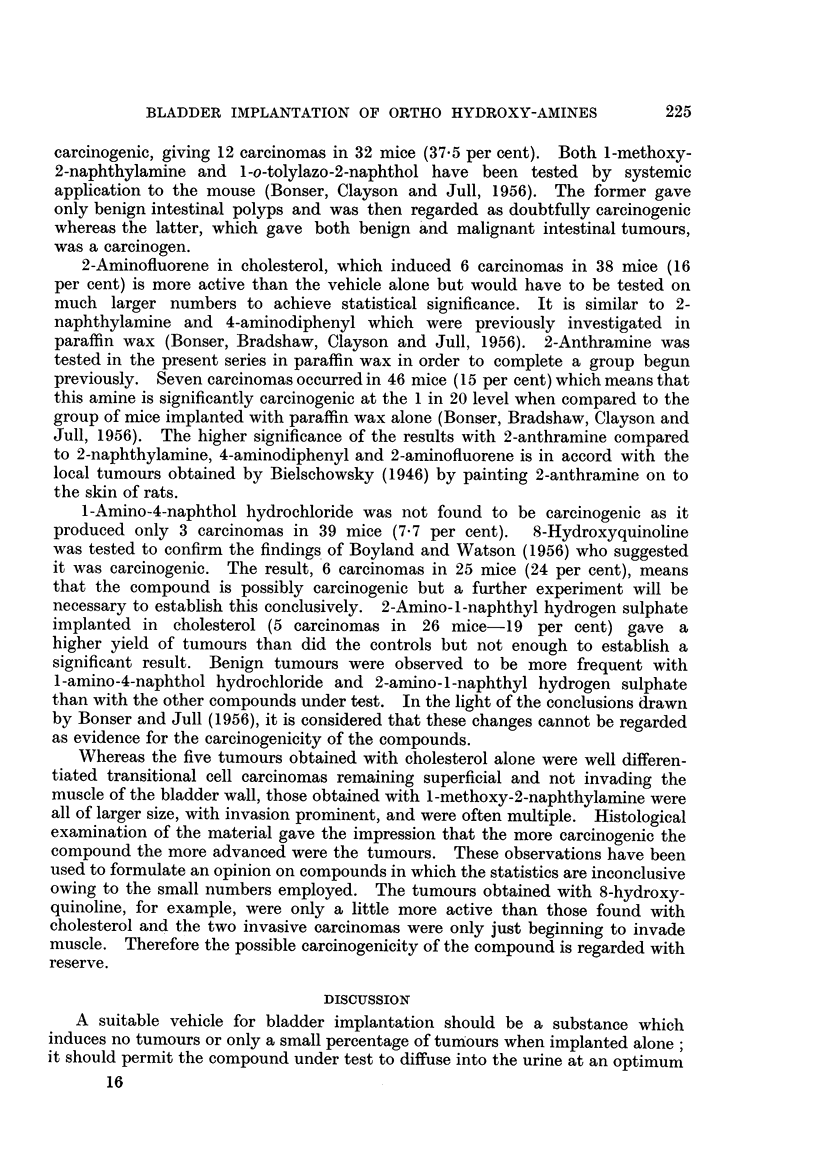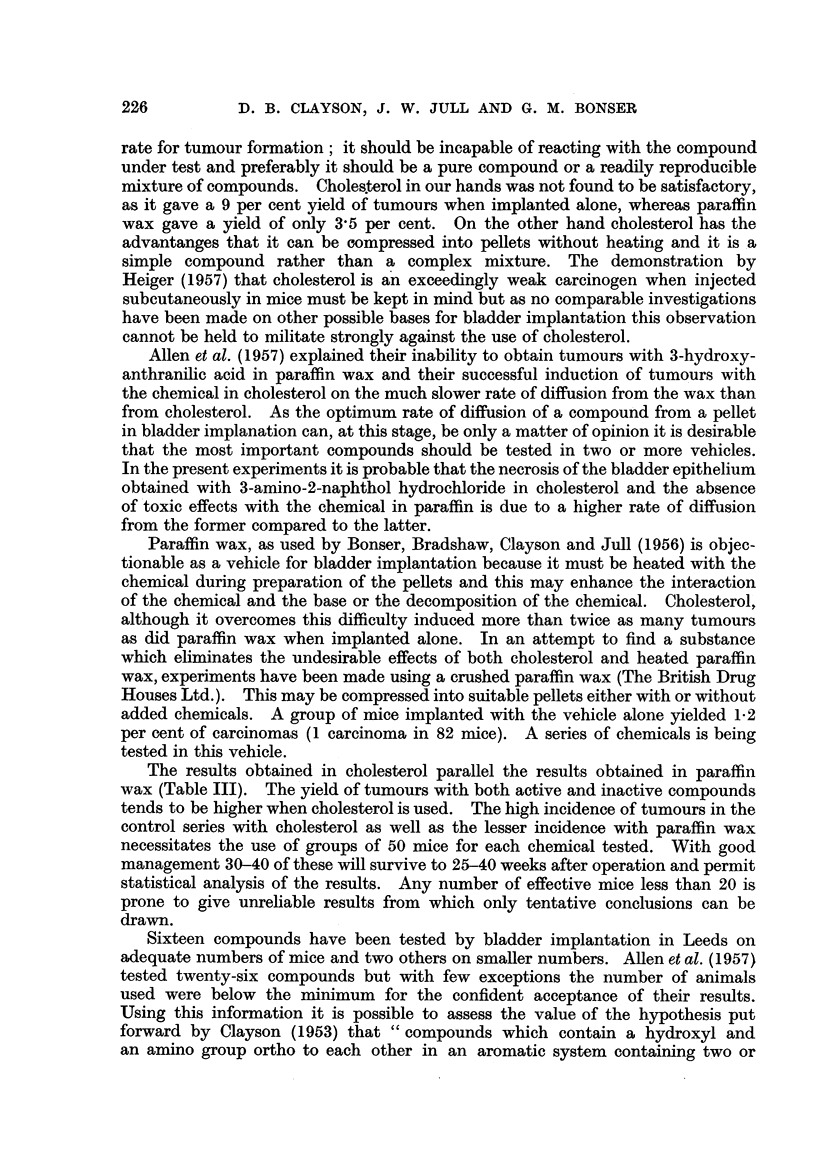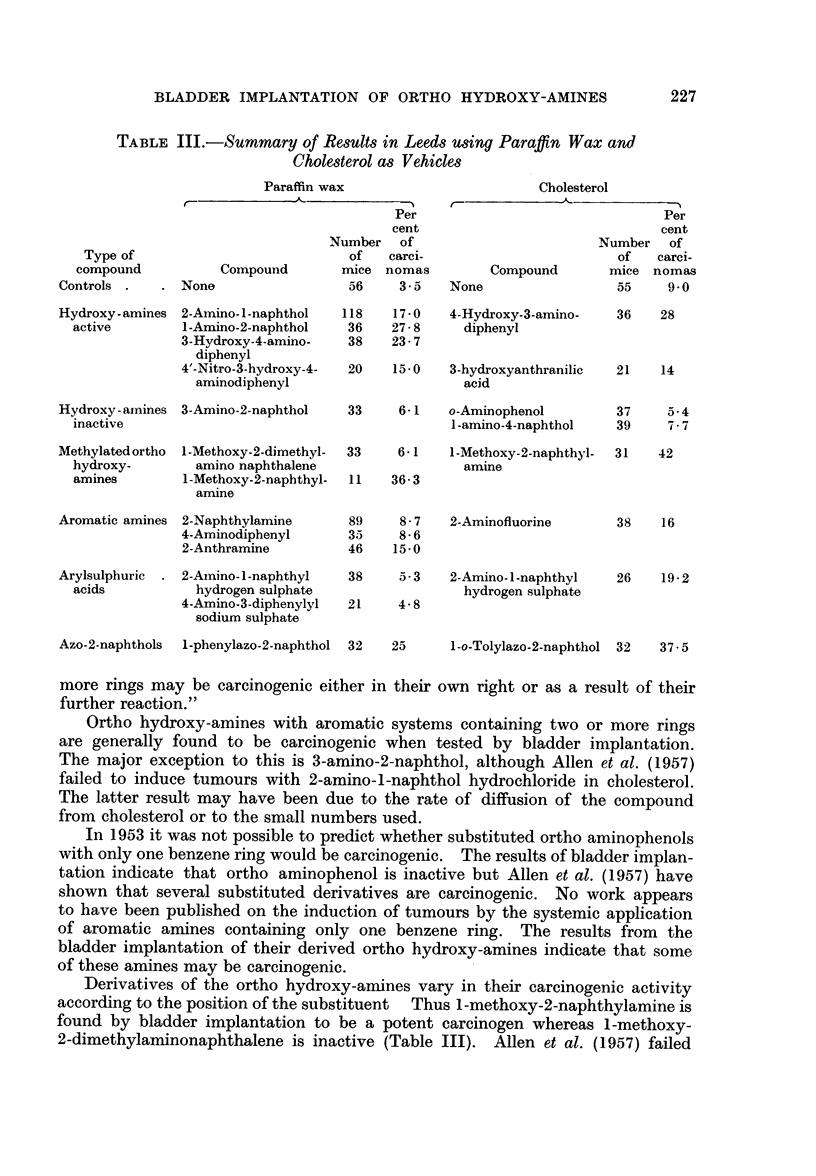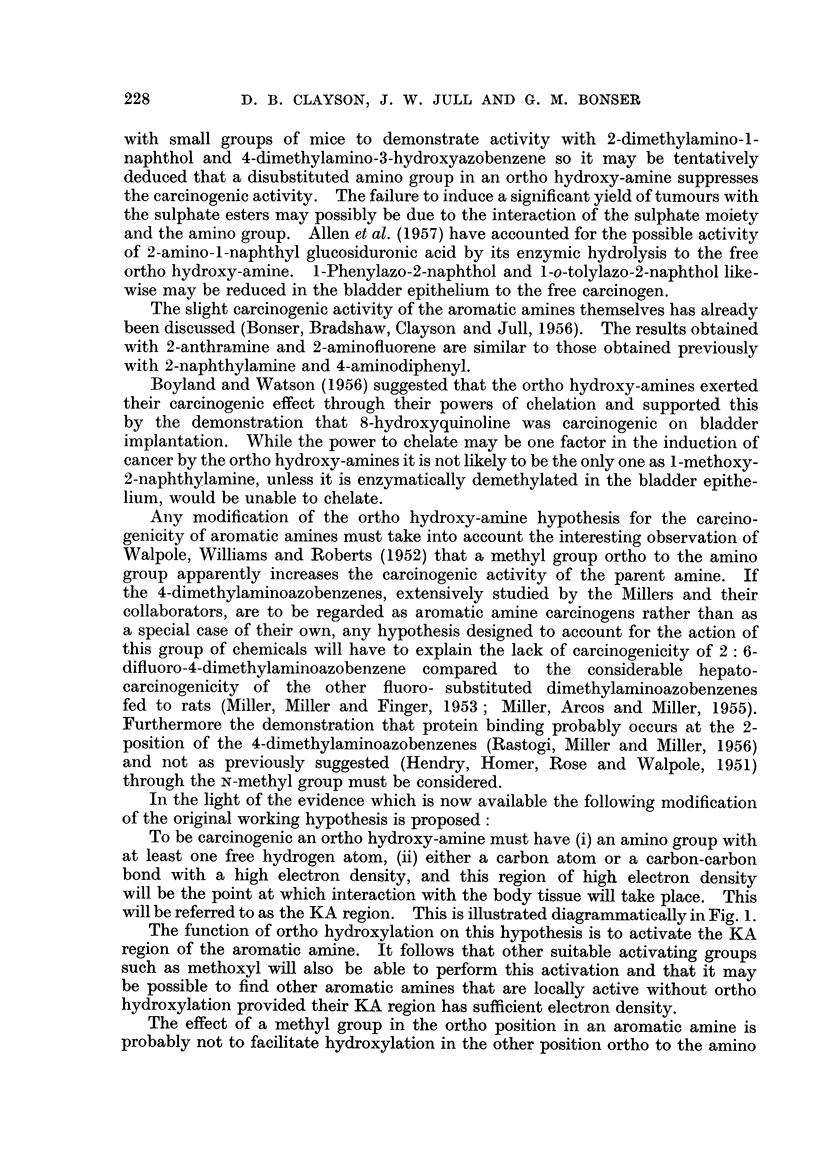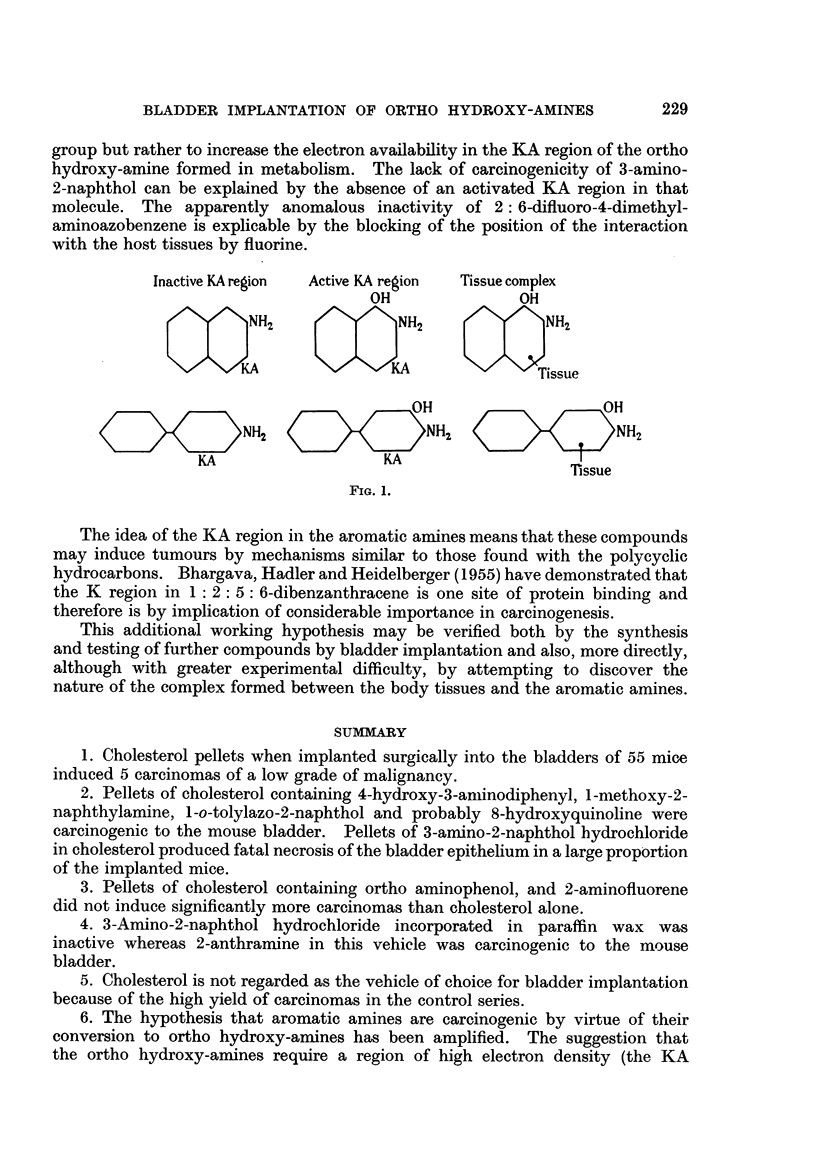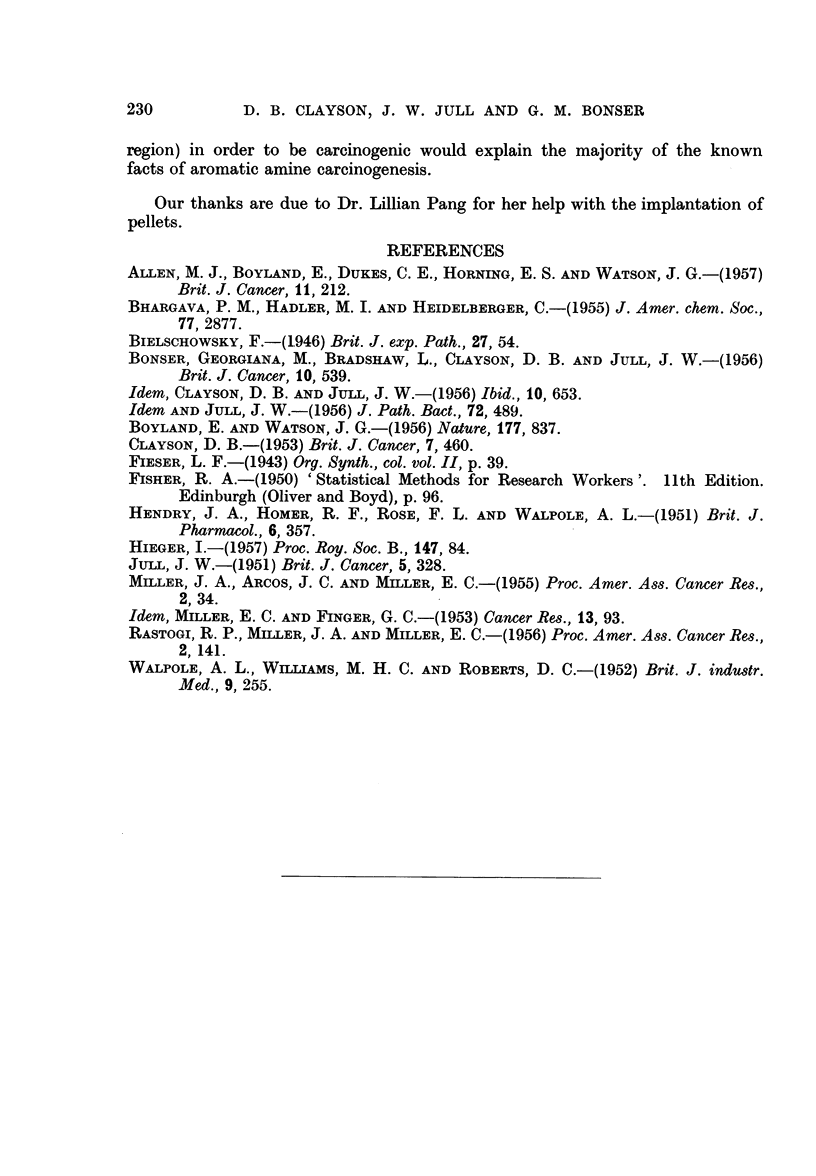# The Testing of Ortho Hydroxy-Amines and Related Compounds by Bladder Implantation and a Discussion of their Structural Requirements for Carcinogenic Activity

**DOI:** 10.1038/bjc.1958.26

**Published:** 1958-06

**Authors:** D. B. Clayson, J. W. Jull, G. M. Bonser


### THE TESTING OF ORTHO HYDROXY-AMINES AND RELATED COMPOUNDS BY BLADDER IMPLANTATION AND A DISCUSSION OF THEIR STRUCTURAL REQUIREMENTS FOR CARCINOGENIC ACTIVITY

D. B. CLAYSON, J. W. JULL AND G. M. BONSER

*From the Department of Experimental Pathology and Cancer Research, University of Leeds*

Received for publication February 27, 1958

BONSER, BRADSHAW, CLAYSON AND JULL (1956) used the method of the surgical implantation of paraffin wax pellets containing added chemicals into the lumen of the mouse bladder to test a series of compounds for carcinogenic activity. The results showed that although pellets containing the aromatic amines, 2-naphthylamine and 4-aminodiphenyl, induced a slightly higher incidence of tumours than did the vehicle alone, the derived ortho hydroxy-amines, 2-amino-1-naphthol hydrochloride and 3-hydroxy-4-aminodiphenyl sulphate, induced a significantly higher yield of tumours. The results from the other compounds tested supported the hypothesis (Clayson, 1953) that the ortho hydroxy-amines are carcinogenic metabolites of the aromatic amines.

Bonser, Bradshaw, Clayson and Jull (1956) used paraffin wax as the base for their pellets, whereas Boyland and Watson (1956) and Allen, Boyland, Dukes, Horning and Watson (1957) used cholesterol and suggested that it was preferable. The purpose of the present experiments was firstly to investigate further the use of cholesterol as a base for bladder implantation and secondly to test additional compounds for carcinogenic activity by this method. As a consequence of these results it has been possible to suggest an amplification of the ortho hydroxy-amine hypothesis for the mode of action of the aromatic amines.

### MATERIALS AND METHODS

The albino mice used were from the same source as described previously (Bonser, Bradshaw, Clayson and Jull, 1956). The pellets were made by compression from an intimate mixture of cholesterol and the chemical (12·5 per cent) under test and were implanted by the method of Jull (1951) as modified by Allen *et al.* (1957). The results were interpreted by the criteria described by Bonser and Jull (1956). Carcinomas which had not invaded muscle were classified as Grade I ; those which had invaded muscle as Grade II. Carcinogenicity was assessed on the total incidence of carcinomas only. Probabilities of statistical significance were evaluated by the exact method for $2 \times 2$ tables (Fisher, 1950).

*Cholesterol* (Batch No. 634495/451004) and 8-*hydroxyquinoline* (Batch No. 424384/531030) were obtained from The British Drug Houses Ltd.

1-*Amino*-4-*naphthol hydrochloride* and 3-*amino*-2-*naphthol hydrochloride* were obtained from Messrs. L. Light and Company and were purified by the method described for the former compound by Fieser (1943).

1-*o-Tolylazo-2-naphthol* (Oil orange TX) was obtained from Messrs. Pointing Ltd., Hexham, and was of food dye quality.

3-*Hydroxyanthranilic acid* was a generous gift from Professor E. Boyland.

2-*Aminofluorene* m.p. 128°, 2-*anthramine* m.p. 238°, 4-*hydroxy*-3-*aminodiphenyl hydrochloride* (free base m.p. 208°), 1-*methoxy*-2-*naphthylamine hydrochloride*, formerly named O-methyl-2-amino-1-naphthol hydrochloride (Bonser, Clayson and Jull, 1956) (free base m.p. 49°) and 2-amino-1-naphthyl hydrogen sulphate (m.p. 224–5°) were prepared in the laboratory.

*o-Aminophenol* was purified by sublimation. It was obtained as a colourless compound which rapidly acquired an orange tinge in contact with air.

### RESULTS

The survival of the mice used in these experiments was better than that obtained previously. Adoption of the modified operative technique of Allen *et al.* (1957) considerably reduced the post-operative mortality, and improved survival of the mice during the experimental period was achieved by the control of ectromelia by vaccination. Of 430 mice which lived for 25 weeks after operation 16 (3·7 per cent) died or were killed *in extremis* at 25–30 weeks, 4 (0·9 per cent) at 31–34 weeks, 22 (5·1 per cent) at 35–39 weeks, while 388 (90·2 per cent) lived to the full 40 weeks of the experiment. The corresponding percentages found by Bonser, Bradshaw, Clayson and Jull (1956) in a previous experiment were 7·4, 6·3, 7·3 and 79. As will be seen from Table I the difference in the rates of survival with the various chemicals was slight.

TABLE I.—*Survival of Implanted Mice Killed after* 25 *Weeks*

| Experiment | Compound | Number of mice killed (weeks) 25–30 | 31–34 | 35–39 | 40 | Total |
|---|---|---|---|---|---|---|
| 1 | Cholesterol alone | 0 | 0 | 0 | 55 | 55 |
| 2 | *o*-Aminophenol | 0 | 1 | 0 | 36 | 37 |
| 3 | 2-Aminofluorene | 0 | 0 | 0 | 38 | 38 |
| 4 | 1-Amino-4-naphthol hydrochloride | 1 | 0 | 1 | 37 | 39 |
| 5 | 8-Hydroxyquinoline | 1 | 0 | 7 | 17 | 25 |
| 6 | 3-Hydroxyanthranilic acid | 1 | 0 | 0 | 20 | 21 |
| 7 | 3-Amino-4-hydroxydiphenyl hydrochloride | 0 | 0 | 1 | 35 | 36 |
| 8 | 1 - Methoxy - 2 - naphthylamine hydrochloride (Cholesterol) | 1 | 0 | 0 | 30 | 31 |
| 9 | 1-*o*-Tolylazo-2-naphthol | 2 | 0 | 3 | 27 | 32 |
| 10 | 2-Amino-1-naphthyl hydrogen sulphate | 1 | 1 | 0 | 24 | 26 |
| 11 | 2-Anthramine (in wax) | 6 | 0 | 2 | 38 | 46 |
| 12 | 3-Amino-2-naphthol hydrochloride (in wax) | 0 | 2 | 4 | 27 | 33 |
| 13 | 1 - Methoxy - 2 - naphthylamine hydrochloride (in wax) | 3 | 0 | 4 | 4 | 11 |

Five carcinomas in 55 mice (9·0 per cent) were obtained when pellets of cholesterol alone were implanted. This incidence of tumours is more than twice as high as that obtained with paraffin wax although the tumours obtained in the present experiment were of a less advanced nature.

Four ortho hydroxy-amines have been tested (Table II). The simplest of these, ortho aminophenol, gave only 2 carcinomas in 37 mice (5·4 per cent) and is inactive. 3-Amino-4-hydroxydiphenyl hydrochloride, on the other hand,

TABLE II.—*Incidence of Bladder Changes in Implanted Mice*

| Experiment | Compound | Base | Number of mice | Squamous metaplasia | Benign* tumours Number | Benign* tumours P | Carcinomas I | Carcinomas II | Carcinomas Total | Carcinomas P |
|---|---|---|---|---|---|---|---|---|---|---|
| 1 | None | Cholesterol | 55 | 7 | 1 | — | 5 | 0 | 5 | — |
| 2 | *o*-Aminophenol | ,, | 37 | 7 | 0 | — | 0 | 2 | 2 | — |
| 3 | 2-Aminofluorene | ,, | 38 | 6 | 3 | 0·18 | 3 | 3 | 6 | 0·25 |
| 4 | 1 - Amino - 4 - naphthol hydrochloride | ,, | 39 | 4 | 5 | 0·044 | 2 | 1 | 3 | — |
| 5 | 8-Hydroxyquinoline | ,, | 25 | 2 | 0 | — | 4 | 2 | 6 | 0·078 |
| 6 | 3-Hydroxyanthranilic acid | ,, | 21 | 6 | 2 | 0·18 | 1 | 2 | 3 | 0·39 |
| 7 | 3 - Amino - 4 - hydroxydiphenyl hydrochloride | ,, | 36 | 6 | 4 | 0·078 | 5 | 5 | 10 | 0·020 |
| 8 | 1 - Methoxy - 2 - naphthylamine hydrochloride | ,, | 31 | 4 | 1 | 0·59 | 6 | 7 | 13 | 0·00053 |
| 9 | 1-*o*-Tolylazo-2-naphthol | ,, | 32 | 9 | 2 | 0·30 | 7 | 5 | 12 | 0·0019 |
| 10 | 2-Amino-1-naphthyl hydrogen sulphate | ,, | 26 | 7 | 5 | 0·012 | 3 | 2 | 5 | 0·17 |
| — | None | Paraffin wax | 56 | 9 | 3 | — | 1 | 1 | 2 | — |
| 11 | 2-Anthramine | ,, ,, | 46 | 7 | 1 | — | 4 | 3 | 7 | 0·043 |
| 12 | 3 - Amino - 2 - naphthol hydrochloride | ,, ,, | 33 | 1 | 0 | — | 1 | 1 | 2 | 0·48 |
| 13 | 1 - Methoxy - 2 - naphthylamine hydrochloride | ,, ,, | 11 | 1 | 1 | 0·52 | 3 | 1 | 4 | 0·005 |

* Mice with both benign and malignant tumours are classified under carcinomas only. No mouse is recorded more than once.

induced 10 carcinomas in 36 mice (28 per cent) and is active. A small group of mice implanted with 3-hydroxyanthranilic acid in cholesterol gave 3 carcinomas in 21 mice (14 per cent) which suggests that the compound may be active, although a larger group of animals is necessary to substantiate this. This has been provided by Allen *et al.* (1957) who found 8 carcinomas in 40 animals (20 per cent) with this compound. When pellets of 3-amino-2-naphthol hydrochloride in cholesterol were implanted into a group of 50 mice, only 6 survived for longer than 48 hours. A smaller group of animals implanted with the sulphate fared no better. At *post mortem* there was complete necrosis of the bladder epithelium. It was therefore decided to implant the chemical in wax, in which base it was found to be inactive (2 carcinomas in 33 mice—6·1 per cent). Three only of 62 mice implanted with either the hydrochloride or the sulphate in cholesterol lived to 40 weeks ; one of these had a carcinoma invading muscle. Thus it is possible that the inactivity of this compound is due to its slow rate of diffusion from paraffin wax. This does not seem likely as the isomeric ortho aminonaphthols are active when implanted in the same paraffin wax and also because compounds with a very low water solubility such as dibenzcarbazole, methylcholanthrene and 1-phenylazo-2-naphthol induce tumours under similar conditions.

Two ortho hydroxy-amine derivatives were investigated : 1-methoxy-2-naphthylamine is the most active compound tested yielding 13 carcinomas in 31 mice (42 per cent) in cholesterol and 4 carcinomas in 11 mice (36·3 per cent) in paraffin wax. This is in marked contrast to 1-methoxy-2-dimethylamino-naphthalene hydrochloride which was tested in wax and found to be inactive (Bonser, Bradshaw, Clayson and Jull, 1956). 1-*o*-Tolylazo-2-naphthol in cholesterol, like 1-phenylazo-2-naphthol in paraffin wax, was found to be locally carcinogenic, giving 12 carcinomas in 32 mice (37·5 per cent). Both 1-methoxy-2-naphthylamine and 1-*o*-tolylazo-2-naphthol have been tested by systemic application to the mouse (Bonser, Clayson and Jull, 1956). The former gave only benign intestinal polyps and was then regarded as doubtfully carcinogenic whereas the latter, which gave both benign and malignant intestinal tumours, was a carcinogen.

2-Aminofluorene in cholesterol, which induced 6 carcinomas in 38 mice (16 per cent) is more active than the vehicle alone but would have to be tested on much larger numbers to achieve statistical significance. It is similar to 2-naphthylamine and 4-aminodiphenyl which were previously investigated in paraffin wax (Bonser, Bradshaw, Clayson and Jull, 1956). 2-Anthramine was tested in the present series in paraffin wax in order to complete a group begun previously. Seven carcinomas occurred in 46 mice (15 per cent) which means that this amine is significantly carcinogenic at the 1 in 20 level when compared to the group of mice implanted with paraffin wax alone (Bonser, Bradshaw, Clayson and Jull, 1956). The higher significance of the results with 2-anthramine compared to 2-naphthylamine, 4-aminodiphenyl and 2-aminofluorene is in accord with the local tumours obtained by Bielschowsky (1946) by painting 2-anthramine on to the skin of rats.

1-Amino-4-naphthol hydrochloride was not found to be carcinogenic as it produced only 3 carcinomas in 39 mice (7·7 per cent). 8-Hydroxyquinoline was tested to confirm the findings of Boyland and Watson (1956) who suggested it was carcinogenic. The result, 6 carcinomas in 25 mice (24 per cent), means that the compound is possibly carcinogenic but a further experiment will be necessary to establish this conclusively. 2-Amino-1-naphthyl hydrogen sulphate implanted in cholesterol (5 carcinomas in 26 mice—19 per cent) gave a higher yield of tumours than did the controls but not enough to establish a significant result. Benign tumours were observed to be more frequent with 1-amino-4-naphthol hydrochloride and 2-amino-1-naphthyl hydrogen sulphate than with the other compounds under test. In the light of the conclusions drawn by Bonser and Jull (1956), it is considered that these changes cannot be regarded as evidence for the carcinogenicity of the compounds.

Whereas the five tumours obtained with cholesterol alone were well differentiated transitional cell carcinomas remaining superficial and not invading the muscle of the bladder wall, those obtained with 1-methoxy-2-naphthylamine were all of larger size, with invasion prominent, and were often multiple. Histological examination of the material gave the impression that the more carcinogenic the compound the more advanced were the tumours. These observations have been used to formulate an opinion on compounds in which the statistics are inconclusive owing to the small numbers employed. The tumours obtained with 8-hydroxyquinoline, for example, were only a little more active than those found with cholesterol and the two invasive carcinomas were only just beginning to invade muscle. Therefore the possible carcinogenicity of the compound is regarded with reserve.

### DISCUSSION

A suitable vehicle for bladder implantation should be a substance which induces no tumours or only a small percentage of tumours when implanted alone ; it should permit the compound under test to diffuse into the urine at an optimum

16

rate for tumour formation ; it should be incapable of reacting with the compound under test and preferably it should be a pure compound or a readily reproducible mixture of compounds. Cholesterol in our hands was not found to be satisfactory, as it gave a 9 per cent yield of tumours when implanted alone, whereas paraffin wax gave a yield of only 3·5 per cent. On the other hand cholesterol has the advantanges that it can be compressed into pellets without heating and it is a simple compound rather than a complex mixture. The demonstration by Heiger (1957) that cholesterol is an exceedingly weak carcinogen when injected subcutaneously in mice must be kept in mind but as no comparable investigations have been made on other possible bases for bladder implantation this observation cannot be held to militate strongly against the use of cholesterol.

Allen *et al.* (1957) explained their inability to obtain tumours with 3-hydroxy-anthranilic acid in paraffin wax and their successful induction of tumours with the chemical in cholesterol on the much slower rate of diffusion from the wax than from cholesterol. As the optimum rate of diffusion of a compound from a pellet in bladder implanation can, at this stage, be only a matter of opinion it is desirable that the most important compounds should be tested in two or more vehicles. In the present experiments it is probable that the necrosis of the bladder epithelium obtained with 3-amino-2-naphthol hydrochloride in cholesterol and the absence of toxic effects with the chemical in paraffin is due to a higher rate of diffusion from the former compared to the latter.

Paraffin wax, as used by Bonser, Bradshaw, Clayson and Jull (1956) is objectionable as a vehicle for bladder implantation because it must be heated with the chemical during preparation of the pellets and this may enhance the interaction of the chemical and the base or the decomposition of the chemical. Cholesterol, although it overcomes this difficulty induced more than twice as many tumours as did paraffin wax when implanted alone. In an attempt to find a substance which eliminates the undesirable effects of both cholesterol and heated paraffin wax, experiments have been made using a crushed paraffin wax (The British Drug Houses Ltd.). This may be compressed into suitable pellets either with or without added chemicals. A group of mice implanted with the vehicle alone yielded 1·2 per cent of carcinomas (1 carcinoma in 82 mice). A series of chemicals is being tested in this vehicle.

The results obtained in cholesterol parallel the results obtained in paraffin wax (Table III). The yield of tumours with both active and inactive compounds tends to be higher when cholesterol is used. The high incidence of tumours in the control series with cholesterol as well as the lesser incidence with paraffin wax necessitates the use of groups of 50 mice for each chemical tested. With good management 30–40 of these will survive to 25–40 weeks after operation and permit statistical analysis of the results. Any number of effective mice less than 20 is prone to give unreliable results from which only tentative conclusions can be drawn.

Sixteen compounds have been tested by bladder implantation in Leeds on adequate numbers of mice and two others on smaller numbers. Allen *et al.* (1957) tested twenty-six compounds but with few exceptions the number of animals used were below the minimum for the confident acceptance of their results. Using this information it is possible to assess the value of the hypothesis put forward by Clayson (1953) that " compounds which contain a hydroxyl and an amino group ortho to each other in an aromatic system containing two or

TABLE III.—*Summary of Results in Leeds using Paraffin Wax and Cholesterol as Vehicles*

| | Paraffin wax | | | Cholesterol | | |
|---|---|---|---|---|---|---|
| Type of compound | Compound | Number of mice | Per cent of carcinomas | Compound | Number of mice | Per cent of carcinomas |
| Controls . . | None | 56 | 3·5 | None | 55 | 9·0 |
| Hydroxy-amines active | 2-Amino-1-naphthol | 118 | 17·0 | 4-Hydroxy-3-amino-diphenyl | 36 | 28 |
| | 1-Amino-2-naphthol | 36 | 27·8 | | | |
| | 3-Hydroxy-4-amino-diphenyl | 38 | 23·7 | | | |
| | 4′-Nitro-3-hydroxy-4-aminodiphenyl | 20 | 15·0 | 3-hydroxyanthranilic acid | 21 | 14 |
| Hydroxy-amines inactive | 3-Amino-2-naphthol | 33 | 6·1 | *o*-Aminophenol | 37 | 5·4 |
| | | | | 1-amino-4-naphthol | 39 | 7·7 |
| Methylated ortho hydroxy-amines | 1-Methoxy-2-dimethyl-amino naphthalene | 33 | 6·1 | 1-Methoxy-2-naphthyl-amine | 31 | 42 |
| | 1-Methoxy-2-naphthyl-amine | 11 | 36·3 | | | |
| Aromatic amines | 2-Naphthylamine | 89 | 8·7 | 2-Aminofluorine | 38 | 16 |
| | 4-Aminodiphenyl | 35 | 8·6 | | | |
| | 2-Anthramine | 46 | 15·0 | | | |
| Arylsulphuric . acids | 2-Amino-1-naphthyl hydrogen sulphate | 38 | 5·3 | 2-Amino-1-naphthyl hydrogen sulphate | 26 | 19·2 |
| | 4-Amino-3-diphenylyl sodium sulphate | 21 | 4·8 | | | |
| Azo-2-naphthols | 1-phenylazo-2-naphthol | 32 | 25 | 1-*o*-Tolylazo-2-naphthol | 32 | 37·5 |

more rings may be carcinogenic either in their own right or as a result of their further reaction."

Ortho hydroxy-amines with aromatic systems containing two or more rings are generally found to be carcinogenic when tested by bladder implantation. The major exception to this is 3-amino-2-naphthol, although Allen *et al.* (1957) failed to induce tumours with 2-amino-1-naphthol hydrochloride in cholesterol. The latter result may have been due to the rate of diffusion of the compound from cholesterol or to the small numbers used.

In 1953 it was not possible to predict whether substituted ortho aminophenols with only one benzene ring would be carcinogenic. The results of bladder implantation indicate that ortho aminophenol is inactive but Allen *et al.* (1957) have shown that several substituted derivatives are carcinogenic. No work appears to have been published on the induction of tumours by the systemic application of aromatic amines containing only one benzene ring. The results from the bladder implantation of their derived ortho hydroxy-amines indicate that some of these amines may be carcinogenic.

Derivatives of the ortho hydroxy-amines vary in their carcinogenic activity according to the position of the substituent Thus 1-methoxy-2-naphthylamine is found by bladder implantation to be a potent carcinogen whereas 1-methoxy-2-dimethylaminonaphthalene is inactive (Table III). Allen *et al.* (1957) failed with small groups of mice to demonstrate activity with 2-dimethylamino-1-naphthol and 4-dimethylamino-3-hydroxyazobenzene so it may be tentatively deduced that a disubstituted amino group in an ortho hydroxy-amine suppresses the carcinogenic activity. The failure to induce a significant yield of tumours with the sulphate esters may possibly be due to the interaction of the sulphate moiety and the amino group. Allen *et al.* (1957) have accounted for the possible activity of 2-amino-1-naphthyl glucosiduronic acid by its enzymic hydrolysis to the free ortho hydroxy-amine. 1-Phenylazo-2-naphthol and 1-*o*-tolylazo-2-naphthol likewise may be reduced in the bladder epithelium to the free carcinogen.

The slight carcinogenic activity of the aromatic amines themselves has already been discussed (Bonser, Bradshaw, Clayson and Jull, 1956). The results obtained with 2-anthramine and 2-aminofluorene are similar to those obtained previously with 2-naphthylamine and 4-aminodiphenyl.

Boyland and Watson (1956) suggested that the ortho hydroxy-amines exerted their carcinogenic effect through their powers of chelation and supported this by the demonstration that 8-hydroxyquinoline was carcinogenic on bladder implantation. While the power to chelate may be one factor in the induction of cancer by the ortho hydroxy-amines it is not likely to be the only one as 1-methoxy-2-naphthylamine, unless it is enzymatically demethylated in the bladder epithelium, would be unable to chelate.

Any modification of the ortho hydroxy-amine hypothesis for the carcinogenicity of aromatic amines must take into account the interesting observation of Walpole, Williams and Roberts (1952) that a methyl group ortho to the amino group apparently increases the carcinogenic activity of the parent amine. If the 4-dimethylaminoazobenzenes, extensively studied by the Millers and their collaborators, are to be regarded as aromatic amine carcinogens rather than as a special case of their own, any hypothesis designed to account for the action of this group of chemicals will have to explain the lack of carcinogenicity of 2 : 6-difluoro-4-dimethylaminoazobenzene compared to the considerable hepatocarcinogenicity of the other fluoro- substituted dimethylaminoazobenzenes fed to rats (Miller, Miller and Finger, 1953 ; Miller, Arcos and Miller, 1955). Furthermore the demonstration that protein binding probably occurs at the 2-position of the 4-dimethylaminoazobenzenes (Rastogi, Miller and Miller, 1956) and not as previously suggested (Hendry, Homer, Rose and Walpole, 1951) through the N-methyl group must be considered.

In the light of the evidence which is now available the following modification of the original working hypothesis is proposed :

To be carcinogenic an ortho hydroxy-amine must have (i) an amino group with at least one free hydrogen atom, (ii) either a carbon atom or a carbon-carbon bond with a high electron density, and this region of high electron density will be the point at which interaction with the body tissue will take place. This will be referred to as the KA region. This is illustrated diagrammatically in Fig. 1.

The function of ortho hydroxylation on this hypothesis is to activate the KA region of the aromatic amine. It follows that other suitable activating groups such as methoxyl will also be able to perform this activation and that it may be possible to find other aromatic amines that are locally active without ortho hydroxylation provided their KA region has sufficient electron density.

The effect of a methyl group in the ortho position in an aromatic amine is probably not to facilitate hydroxylation in the other position ortho to the amino group but rather to increase the electron availability in the KA region of the ortho hydroxy-amine formed in metabolism. The lack of carcinogenicity of 3-amino-2-naphthol can be explained by the absence of an activated KA region in that molecule. The apparently anomalous inactivity of 2 : 6-difluoro-4-dimethyl-aminoazobenzene is explicable by the blocking of the position of the interaction with the host tissues by fluorine.

FIG. 1.

The idea of the KA region in the aromatic amines means that these compounds may induce tumours by mechanisms similar to those found with the polycyclic hydrocarbons. Bhargava, Hadler and Heidelberger (1955) have demonstrated that the K region in 1 : 2 : 5 : 6-dibenzanthracene is one site of protein binding and therefore is by implication of considerable importance in carcinogenesis.

This additional working hypothesis may be verified both by the synthesis and testing of further compounds by bladder implantation and also, more directly, although with greater experimental difficulty, by attempting to discover the nature of the complex formed between the body tissues and the aromatic amines.

SUMMARY

1. Cholesterol pellets when implanted surgically into the bladders of 55 mice induced 5 carcinomas of a low grade of malignancy.

2. Pellets of cholesterol containing 4-hydroxy-3-aminodiphenyl, 1-methoxy-2-naphthylamine, 1-*o*-tolylazo-2-naphthol and probably 8-hydroxyquinoline were carcinogenic to the mouse bladder. Pellets of 3-amino-2-naphthol hydrochloride in cholesterol produced fatal necrosis of the bladder epithelium in a large proportion of the implanted mice.

3. Pellets of cholesterol containing ortho aminophenol, and 2-aminofluorene did not induce significantly more carcinomas than cholesterol alone.

4. 3-Amino-2-naphthol hydrochloride incorporated in paraffin wax was inactive whereas 2-anthramine in this vehicle was carcinogenic to the mouse bladder.

5. Cholesterol is not regarded as the vehicle of choice for bladder implantation because of the high yield of carcinomas in the control series.

6. The hypothesis that aromatic amines are carcinogenic by virtue of their conversion to ortho hydroxy-amines has been amplified. The suggestion that the ortho hydroxy-amines require a region of high electron density (the KA

region) in order to be carcinogenic would explain the majority of the known facts of aromatic amine carcinogenesis.

Our thanks are due to Dr. Lillian Pang for her help with the implantation of pellets.

### REFERENCES

ALLEN, M. J., BOYLAND, E., DUKES, C. E., HORNING, E. S. AND WATSON, J. G.—(1957) *Brit. J. Cancer*, **11**, 212.

BHARGAVA, P. M., HADLER, M. I. AND HEIDELBERGER, C.—(1955) *J. Amer. chem. Soc.*, **77**, 2877.

BIELSCHOWSKY, F.—(1946) *Brit. J. exp. Path.*, **27**, 54.

BONSER, GEORGIANA, M., BRADSHAW, L., CLAYSON, D. B. AND JULL, J. W.—(1956) *Brit. J. Cancer*, **10**, 539.

*Idem*, CLAYSON, D. B. AND JULL, J. W.—(1956) *Ibid.*, **10**, 653.

*Idem* AND JULL, J. W.—(1956) *J. Path. Bact.*, **72**, 489.

BOYLAND, E. AND WATSON, J. G.—(1956) *Nature*, **177**, 837.

CLAYSON, D. B.—(1953) *Brit. J. Cancer*, **7**, 460.

FIESER, L. F.—(1943) *Org. Synth., col. vol. II*, p. 39.

FISHER, R. A.—(1950) 'Statistical Methods for Research Workers'. 11th Edition. Edinburgh (Oliver and Boyd), p. 96.

HENDRY, J. A., HOMER, R. F., ROSE, F. L. AND WALPOLE, A. L.—(1951) *Brit. J. Pharmacol.*, **6**, 357.

HIEGER, I.—(1957) *Proc. Roy. Soc.* B., **147**, 84.

JULL, J. W.—(1951) *Brit. J. Cancer*, **5**, 328.

MILLER, J. A., ARCOS, J. C. AND MILLER, E. C.—(1955) *Proc. Amer. Ass. Cancer Res.*, **2**, 34.

*Idem*, MILLER, E. C. AND FINGER, G. C.—(1953) *Cancer Res.*, **13**, 93.

RASTOGI, R. P., MILLER, J. A. AND MILLER, E. C.—(1956) *Proc. Amer. Ass. Cancer Res.*, **2**, 141.

WALPOLE, A. L., WILLIAMS, M. H. C. AND ROBERTS, D. C.—(1952) *Brit. J. industr. Med.*, **9**, 255.